# Ergotin

**Published:** 1910-04

**Authors:** H. H. Redfield

**Affiliations:** Chicago, Ill., Professor of Therapeutics, Illinois Medical College; Department of Medicine, Loyola University, Chicago, Illinois, Professor of Therapeutics and Physiology, Reliance Medical College, Chicago, Illinois


For Texas Medical Journal.

### Ergotin.

BY H. H. REDFIELD, M. D., CHICAGO, ILL.,

Professor of Therapeutics, Illinois Medical College; Department of Medicine, Loyola University, Chicago, Illinois. Professor of Therapeutics and Physiology, Reliance Medical College, Chicago, Illinois.

Ergotin is the purified aqueous extract of ergot, and represents the full medicinal value of that plant. The standard granule gr. 1-6 represents about six drops of the fluid extract.

### PHYSIOLOGICAL ACTION.

Ergotin is classed as a motor-excitant, vaso-constrictor, cardiac sedative, and stimulant of involuntary muscle tissue. It is also a hemostatic, ecbolic, anhidrotic and convulsant.

The exhibition of ergotin in large doses at first produces a temporary fall in blood-pressure, which is due to the depressing action of the drug on the heart; soon, however, the vaso-constrictor effect of the drug manifests itself with a resultant general constriction of the blood-vessels throughout the entire body, and a consequent rise in blood pressure, an arterial ischemia being produced owing to the blood supply being decreased.

Regarding the effect of ergotin, considerable controversy has arisen, some physiologists claiming that the action is the result of a combined stimulation of the vaso-constrictor center in the medulla, and the unstriped fibers in the walls of the blood-vessels; while other equally careful and competent observers contend that it is due entirely to an action on the vaso-motor center alone.

In excessive doses ergotin has a depressing action upon both the heart and vaso-motor center. The primary fall of blood pressure which this drug produces is not reversed as it is in the case of moderate doses, but continues, and as a result a paralysis, progressive in nature, of the heart and vaso-motor apparatus is produced.

By its stimulating action on the centers in the cord which govern the uterine muscle tissue, ergotin produces powerful contractions of the uterus, especially the parturient uterus. On the impregnated but not parturient uterus this action is not so constant, and though the exhibition of ergotin often produces abortion, yet in many instances it fails utterly to initiate uterine contractions in pregnant females.

Its action as a constrictor of the blood-vessels makes it a valuable agent in controlling post-partum hemorrhage, when it practically seals up the hemorrhagic points in the walls of the uterus, notwithstanding the increased blood pressure in the vessels.

"The phenomena produced by ergotin are divided into two classes, according as the drug is taken in large quantity for a short time, or in small doses for a considerable period. In large doses it acts as a gastro-intestinal irritant, causing nausea and vomiting, colic, thirst and purging. It slows the heart, raises arterial tension greatly, dilates the pupils, and produces pallor and vertigo with frontal headache. It stimulates the contractions of unstriped muscle fiber, especially affecting the sphincters and causing contraction of the sphincter of the bladder, making micturition difficult if not impossible. It produces cerebral and spinal anemia, a great fall of the body temperature, coldness of the surface, tetanic spasms, and violent convulsions. A very large dose is necessary to produce these results, and as much as three ounces of the fluid extract of ergot have been given daily for a week or more without producing any marked effect" (Potter).

### THERAPEUTICS.

Ergotin is indicated in women who are thin, emaciated, cachetic, of a melancholy turn of mind, who complain of a constant bearing down sensation in the uterus, and who, as a rule, suffer more or less from passive hemorrhage.

In conjunctivitis and general inflammations of the mucous membranes the local application or internal administration of ergot is highly beneficial.

It should be remembered in headache of a congestive nature, when the pain begins at the back of the neck and extends over the entire occiput and head; the extremities are cold and livid, the patient much distressed and pale, and the face wears an extremely anxious expression.

Incontinence of urine when due to a lax condition of the sphincter of the bladder is quickly corrected by ergotin.

Headaches occurring during the climacteric period, when the feet and hands are cold and clammy, the face pale and drawn, derive marked benefit from the exhibition of ergotin.

It should be studied in diarrhea when the skin is shriveled and covered with a cold, clammy sweat; the patient though feeling cold objects to being covered up, and will throw off the bed clothing. Sometimes there is involuntary passage of the stools; aneuria is present; the abdomen is tympanitic, and the patient greatly prostrated.

Ergotin, by slowing the rate of the flow in the blood-vessels, is of great benefit in hastening or aiding coagulation in aneurism.

It should be kept in mind in cases of spinal meningitis.

According to Da Costa, the internal administration of ergotin will markedly reduce an enlarged spleen.

It is of service in those cases of impotence due to an escape of blood from the dorsal vein of the penis, which makes erection an almost impossible matter.

In mania produced by cerebral anemia, ergotin is very useful.

Compare its action with that of arsenic and colchicine.

